# Stakeholder arguments during the adoption of a sugar sweetened beverage tax in South Africa and their influence: a content analysis

**DOI:** 10.1080/16549716.2022.2152638

**Published:** 2022-12-12

**Authors:** Safura Abdool Karim, Petronell Kruger, Natasha Mazonde, Agnes Erzse, Susan Goldstein, Karen Hofman

**Affiliations:** a SAMRC Wits Centre for Health Economics and Decision Science (PRICELESS SA), Wits School of Public Health, University of Witwatersrand, Johannesburg, South Africa; b College of Law and Management Studies, University of KwaZulu-Natal, Durban, South Africa

**Keywords:** Obesity, diet-relatednon-communicable diseases, non-communicable diseases, sugary beverages, fiscal policy

## Abstract

**Background:**

Sugar-sweetened beverage (SSB) taxes are recognised as an effective intervention to prevent obesity. More countries are adopting SSB taxes, but the process of the adoption is politically complex.

**Objective:**

This study aimed to analyse how public participation processes influenced the South African tax.

**Methods:**

We conducted a content analysis of documents associated with the process of adopting the tax. Records were identified utilising the Parliamentary Monitoring Group database, including draft bills, meeting minutes and written submissions. The records were categorised and then inductively coded to identify themes and arguments.

**Results:**

We identified six cross-cutting themes advanced by stakeholders: economic considerations, impact on the vulnerable, responsiveness of an SSB tax to the problem of obesity, appropriateness of an SSB tax in South Africa, procedural concerns, and structure of the tax. Stakeholder views and arguments about the tax diverged based on their vested interests. The primary policymaker was most responsive to arguments concerning the economic impact of a tax, procedural concerns and the structure of the tax, reducing the effective rate to address industry concerns.

**Conclusion:**

Both supportive and opposing stakeholders influenced the tax. Economic arguments had a significant impact. Arguments in South Africa broadly echoed arguments advanced in many other jurisdictions.

## Introduction

There is a growing burden from diet-related non-communicable diseases (NCDs) in sub-Saharan Africa (SSA) [1]. Like many countries in SSA, South Africa has an increasing burden of obesity and associated NCDs. A 2018 study showed high levels of overweight and obesity, particularly in black women in Soweto, South Africa [2]. The characteristics of the obesity epidemic are complicated by a double burden of malnutrition in South Africa, manifesting even at a household-level where overweight or obese mothers have undernourished children [3]. Consequently there is a need to address obesity and prevent the associated diseases.

There is increasing evidence that diet-related NCDs diseases may be prevented through the implementation of various population level interventions aimed at improving diet and nutrition [4,5]. More countries are adopting SSB taxes as a measure which contributes to the prevention of diet-related NCDs [5].

Although SSB taxation is an evidence-based intervention, adoption and implementation remain politically complex [6–9]. Governments seeking to adopt SSB taxation often face intense opposition from commercial, and even government, actors [6,8,10,11]. In SSA, particular, the public health benefits of SSB taxation may conflict with aspirations of economic growth [7,10]. This can prevent the adoption of policies, as in Colombia, or lead to weaker or diluted policies [6,7,12,13]. These difficulties may be compounded in countries, such as South Africa where the sugar and SSB industry have historically or currently contribute to the economy [14]. In particular, South Africa has become an entry point into Africa for many multi-national SSB producers and thus measures targeting SSBs would be anticipated to face opposition from both beverage and sugar producers [15].

South Africa’s Health Promotion Levy (HPL) was the first African public health SSB tax when adopted on 1 April 2018. The process of adopting the tax was complex. Civil society, academics and government actors supported the tax, buttressed by context-specific local evidence [16]. Industry and labour unions strongly opposed to tax, citing potential job losses and economic harms [10]. Public views were mixed with Boisire et al’s indicated that consumers were cynical about the government’s motives in adopting the tax [17]; while other research showed broad public support for the tax [18].

The formal policymaking process offers a means to understand how different stakeholders respond to and influence SSB taxes. Analysis of submissions on the New York soda portion policy described the viewpoints of different actors and their arguments supporting or opposing the policy [19]. In Brazil and South Africa, analysis of the policymaking processes provided a way to understand context-specific responses to SSB taxes of value in similar contexts [16,20]. However, the effect different stakeholders had on the adoption of an SSB tax and their framing of the policy has not been considered yet.

This study seeks to address this gap by using data from the formal policymaking process to understand stakeholders’ framing and influence on SSB taxes in South Africa.

## Methods

The study design was based on Roberto and Pomeranz’s content analysis of public submissions [19]. We supplemented this with analysis of policymaker responses to the SSB tax to describe the formal policymaking process and influence exerted by different actors. The study sought to (1) map the formal policymaking process, (2) describe stakeholder arguments and (3) analyse how the key policymaker, Treasury, responded to these arguments.

### Data collection

**Figure 1.**
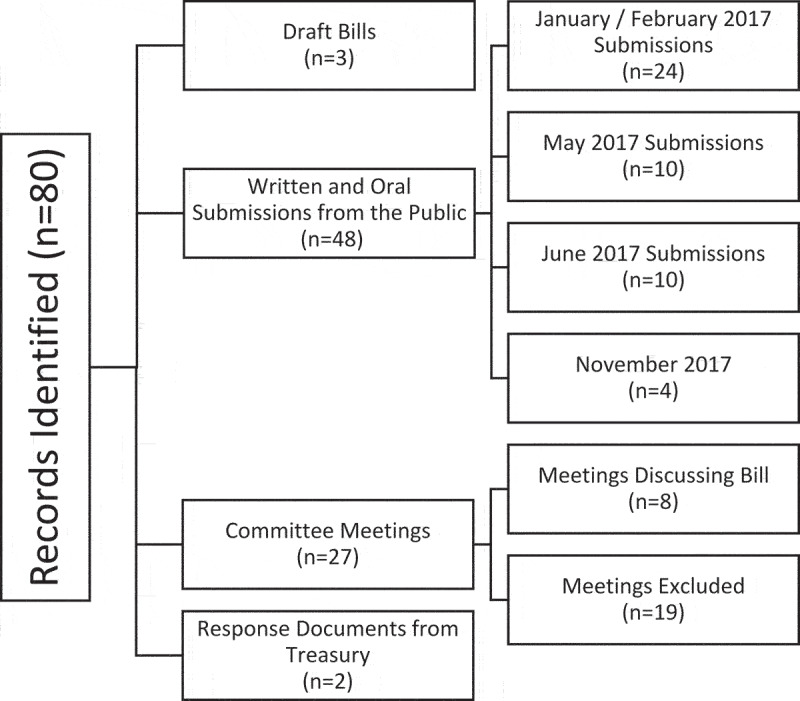
Record selection process.

We utilised the Parliamentary Monitoring Group database [21], a digital, open-access database, to obtain written and audio records of activities related to the SSB tax. We searched the database using the terms ‘sugar tax’, ‘sugary beverage tax’, ‘sugar-sweetened beverage tax’ and ‘health promotion levy’ dated from February 2016, when the tax was announced, to April 2018, when it was implemented. We also utilised the Bill Tracker feature to collect data on the form of legislative process. Types of records identified through this search included public hearings and written submissions, committee meetings minutes and responses from Treasury. Documents included in the final analysis were 3 draft bills, 5 public hearings, 27 committee meeting minutes, 48 written submissions and 2 response documents from Treasury (Figure 1). The documents were then manually reviewed for relevance. This consisted of two researchers reviewing documents to check whether SSB taxes or the HPL were discussed and excluding documents where the terms were mentioned but not substantively dealt with. As a result of this screening 18 meeting minutes were excluded after the initial review as irrelevant, with the remaining 9 being included in the analysis.

### Data analysis

SAK and PKdownloaded and reviewed both written and audio records. From this review, a timeline for the tax was constructed. This timeline and process was compared to the processes followed for other taxes adopted in the same period to identify where the SSB tax process deviated. Draft bills and policy documents were coded deductively using a pre-defined codebook developed to identify the features of the tax including scope and structure of the tax over the period. We then inductively coded the submissions and meeting minutes for their arguments and content. Two researchers doubled coded 10% of the submissions using the pre-defined codebook and also inductively coded themes and arguments to develop the codebook following the approach adopted by Roberto and Pomeranz [19]. Thereafter the entire research team met to validate the overarching themes and refine the final codebook. The research team then met twice, first to group the different arguments and content into broader themes as well as by stakeholders and then to validate the findings. We discussed discrepancies, refined and finalised the codebook. During the analysis different arguments and content were grouped into broader themes as well as by stakeholders. The themes were then used to deductively code the Treasury response documents.

### Ethics

This study used publicly available information, and was conducted under an ethics waiver from the University of the Witwatersrand Human Research Ethics Committee (clearance certificate no. HRECNMW20/07/05). Despite the public nature of the documents, we elected to adopt a level of anonymisation to the information in light of the fact that we did not obtain explicit consent from the persons and/or stakeholders who made the submissions. We acknowledge that the availability of information through the internet may lead to complex ethical issues, particularly in the context of publicly posted content and thus have adopted a cautious stance.

## Results

### Overview of the parliamentary process

**Figure 2.**
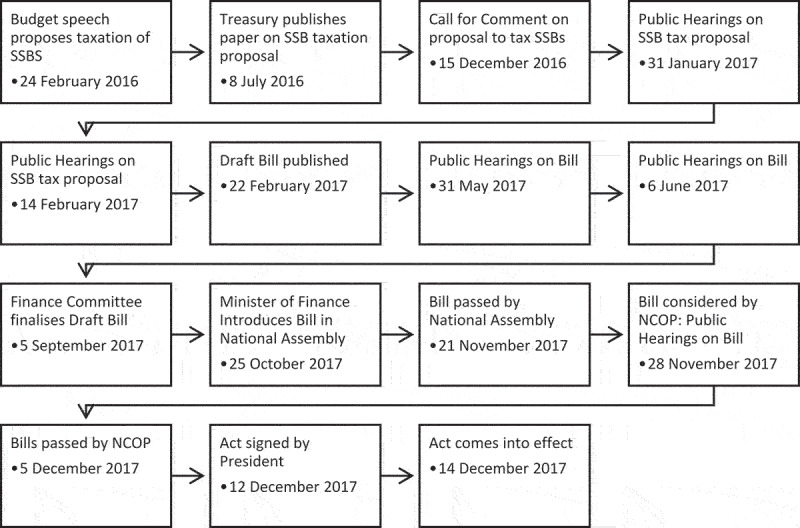
Timeline of the parliamentary process of adopting an SSB tax.

Figure 2 provides a timeline of the policy process of the SSB tax from its announcement in February 2016 to its adoption in December 2017.

The process of passing the HPL was initiated by the announcement of the tax in the 2016 budget speech [22]. On 8 July 2016, National Treasury published a proposal for an SSB tax, which was an excise tax based on added sugar in non-alcoholic beverage and excluded intrinsic sugars [23]. The effective rate of the proposed tax was 20% to be implemented from April 2017. Comments on this proposal are not publicly available.

On 15 December 2016, the Standing Committee on Finance and Portfolio Committee on Health invited comments on a proposed SSB tax not related to the Treasury proposal. The Committee had two public hearings, and Treasury provided a preliminary response document which outlined a proposed tax similar to the July proposal.

In 2017, the Committee published a draft Bill which contained a changed tax structure [24]. The bill introduced an exemption for the first 4 g sugar/100 ml, and the tax rate was lowered from 2.29c to 2.1c per gram of sugar. Concentrates were subject to a lower rate of 1.05c/g of sugar. There were two public hearings held and a number of discussions of the Bill within the Committee.

Before the Bill was finalised and tabled for a vote in the Committee, the tax was amended to tax concentrates at the same rate as other SSBs [24]. However, the sugar content of concentrate was calculated on an ‘as consumed’ basis, i.e. the diluted product, which further reduced the effective tax rate on concentrates. The Committee voted to approve the Bill on 15 September 2017 [24]. The Bill was then passed by National Assembly on 21 November. The Bill was then considered by the National Council of Provinces who, unusually, allowed for further public submissions before passing the Bill. The President promulgated the Health Promotion Levy in December 2017, and in April 2018, it was implemented [24].

### Stakeholder views on SSB taxation

#### Overview

Table 1 provides a an overview of the 48 stakeholder submissions analysed. A majority of submissions (47,9%) were made by industry-related organisations, comprising largely of industry associations, such as sugar and SSB producers. Non-governmental organisations accounted for 21 (43,8%) of submissions, comprising mostly of academia and civil society, such as public health research units and health advocacy organisations. 45,8% (22) of the submissions supported adopting an SSB tax. Of the submissions opposing the tax, 39,6% (19) opposed the adoption of a tax and 12,5% (6) opposed a specific part of proposed tax.

**Table 1.** Overview of submissions on the SSB tax (n = 48).

Submissions	Opposed	Opposed Specific Part	Neutral	Supported	Total
Industry-related organisations	16	6	1	0	23
Industry	5	4	0	0	9
Industry association	10	1	0	0	11
Industry-funded research	1	1	1	0	3
Labour unions	2	0	0	0	2
Non-governmental organizations	1	0	0	20	21
Academic institutions	0	0	0	7	7
Civil society organisations	0	0	0	8	8
Health associations	0	0	0	4	4
Other	1	0	0	1	2
Government departments	0	0	0	2	2
*Total*	*19 (39,6%)*	*6 (12,5%)*	*1 (2,1%)*	*22 (45,8%)*	*48 (100%)*

Nine committee meetings were analysed for the political affiliation of committee members and their stance on the tax. Participation varied across the eight committee meetings (Table 2) with the majority party (African National Congress) and official opposition (Democratic Alliance) having the highest participation with 50–67% and 20–50% representation respectively across meetings. Positions on the tax were not homogenous within parties, with the stances in meetings outlined in Table 2.

**Table 2.** Political parties’ positions on HPL as a % of comments made on the HPL in committee meetings (30 words).

Position on the Tax	Opposed (%)	Opposed specific part (%)	Neutral (%)	Supported (%)
African National Congress	3	3	32	62
Democratic Alliance	0	22	56	22
Economic Freedom Front	0	0	50	50
National Freedom Party	0	0	0	100

#### Stakeholder arguments

Six themes emerged from the qualitative analysis of submissions and committee meetings: economic considerations, impact on the vulnerable, procedural concerns, the responsiveness of an SSB tax to the problem of obesity, the appropriateness of an SSB tax in SA and structure of the tax. Table 3 provides an overview of the key arguments, the stakeholders raising them and the number of instances raised.

**Table 3.** Breakdown of key arguments made on the HPL in submissions.

Theme	Argument	Instances raised	Stakeholders raising argument (n)
Economic considerations	Effect on small and medium enterprises (SMEs) Negative effect on the economy Job losses Economic benefits of the tax	34	Academia (5) Civil Society (6) Industry (5) Industry Associations (11) Health Associations (2) Labour (3) Other (2)
Impact on the vulnerable	Impact on the poor/regressive nature of the tax Impact on children	26	Academia (4) Civil Society (5) Industry (4) Industry Associations (5) Health Associations (4) Labour (3) Other (1)
The responsiveness of an SSB tax to the problem of obesity	Support of government action to reduce obesity Complexity of the problem of obesity Alternative actions to the tax through voluntary measures, public-private partnerships and other existing measures Health benefits of the tax	38	Academia (5) Civil Society (7) Industry (6) Industry Associations (11) Health Associations (4) Labour (3) Other (2)
The appropriateness of an SSB tax in South Africa	Evidence on efficacy, impact and feasibility of the tax Administrative burden of implementing the tax Efficacy of the tax in other countries	43	Academia (7) Civil Society (8) Industry (6) Industry Associations (9) Industry Research (3) Health Associations (5) Labour (3) Other (2)
Procedural concerns	Public engagement Stakeholder and cross-sectoral engagement Jurisdiction Evaluation of socio-economic impact Delay adoption of the tax	23	Academia (2) Civil Society (2) Industry (5) Industry Associations (8) Health Associations (1) Labour (3) Other (2)
Structure of the tax	Scope of the tax Appropriate tax rate Exemptions or threshold Potential earmarking and use of revenue Inclusion of 100% fruit juices and dairy products	33	Academia (7) Civil Society (5) Industry (7) Industry Associations (5) Industry Research (2) Health Associations (3) Labour (2) Other (2)

##### Economic considerations

Economic considerations appeared in 34 of the submissions. Issues raised in opposition to the tax included potential job losses, and negative economic impacts on related sectors and industries. Economic arguments raised in support of the tax argued that the tax would result in healthcare savings.

Industry actors and trade unions argued strongly cautioned that the HPL would lead to job losses, including the risk to jobs within their organisations or companies. These submissions often included providing internally generated estimates on the nationwide impact of the tax as causing over 20,000 job losses. There was emphasis on the negative impact a tax would have on sugar-producers, with the sugar industry arguing that the tax would undermine economic growth in the country.

The potential of sugarcane agricultural land going out of production and the consequent jobs losses, will certainly not support government’s strategic plan of prioritising agriculture for economic growth, revitalisation and job creation. (Industry Association_4)

Supporters of the tax also discussed job losses but contextualised this against public health benefits. calling on government to *‘reject the notion that health must be traded off against the threat of job losses’ (Health Associations_1)* or to challenge industry assertions about the scale of job losses. ‘*To suggest that South Africans must keep dying in vast numbers (34500 annually) to preserve a much smaller number sugar industry jobs is both immoral and wrong.’* (CSO_4)

Academic and civil society also highlighted the economic benefits of the HPL, particularly in relation to the savings in healthcare spend and avoiding economic hardship resulting from NCD morbidity.

##### Impact on vulnerable persons or groups

The impact of an SSB tax on vulnerable groups, specifically the poor, children, and rural dwellers, was raised by both opponents and proponents. Opponents emphasised the negative impact while supporters argued the regressive structure of the tax would yield greater health benefits for poorer consumers. *‘The fact that the SSB tax will disproportionately affect the poor is an important consideration. An argument can be made that this burden may be outweighed by the fact that non-communicable diseases and obesity also disproportionately affect the poor’* (CSO_1).

Opponents of the tax emphasised that poorer consumers relied on sugar and SSBs for their affordability as an energy source: ‘*the hungry and starving in our communities are many and they need affordable energy to be able to live, survive and grow … sugar provides such a source of affordable energy’* (*Industry Association_7)* and *‘[SSBs] are very important in a country and continent with an active mining industry and high temperatures when labourers are working outdoors and cannot drink enough water to combat dehydration’* (Industry Association_7).

Proponents of the tax noted that obesity and NCDs in South Africa disproportionately affected poor women, and as a consequence, these groups would particularly benefit from the implementation of the tax. One CSO group stated that while the SSB tax was economically regressive, it was progressive for health.

##### Responsiveness to obesity

Whether an SSB tax was responsive to the obesity epidemic was an point of contention, with most stakeholders stating that it was necessary to respond to the growing obesity crisis. The point of divergence in this regard was whether fiscal measures such as an SSB tax were suitable obesity-prevention interventions for SA.

Stakeholders from the sugar industry questioned the links between sugar consumption and obesity. *‘No correlation exists between sugar consumption and obesity in SA’* (Industry_1).

Other industry actors argued from voluntary actions and self-regulation to address obesity. *‘Industry’s proposed alternative contemplates a regulated sugar reduction with no tax on sugar sweetened beverages. Binding and measurable health and economic commitments … education campaigns [and] Continue to innovate and provide greater variety of low and no-sugar variants’* (Industry_4).

Both opponents and proponents of the tax argued that the complexity of addressing obesity required additional measures and a comprehensive response. However, opponents of the tax frequently used this argument to undermine the value of an SSB tax, stating, ‘*targeting of an individual ingredient in a particular food product as the tax aims to do, is highly unlikely to resolve a complex health condition that requires a multi-disciplinary approach, including an improvement of the current government health care system*’ (Industry Association_7).

##### Appropriateness in South Africa

The arguments on whether an SSB tax was appropriate for the South African context was raised almost entirely in opposition to the tax. Opponents questioned whether the tax would achieve the intended result and whether it would be too administratively burdensome to implement. The scope of the tax and inclusion of certain products was also challenged.

Scientific evidence on the effectiveness of a tax was utilised by opponents and proponents of the tax. Proponents often cited the success of an SSB tax in Mexico and other countries where evidence showed reduced consumption of SSBs. *‘Recent evidence from Mexico, and Berkeley, California in the USA show that taxing sugary drinks lowers the consumption of these unhealthy beverages, increase the sales and consumption of healthier alternatives, and do not result in revenue losses for businesses or job losses’* (CSO_2).

Opponents raised instances where an SSB tax was ineffective and argued that evidence from countries like Mexico wasn’t transferable to South Africa, ‘*the cultural variances and preferences in South Africa should specifically be considered as to whether SSBs taxation will affect the intended behaviour and choices of saturated sugar consumption to decrease obesity*’ (Industry Association_1). Sugar producers and related industry actors raised the differences between the operation of the sugar market in Mexico and SA.

Some opponents argued that fiscal measures were inappropriate in addressing health issues. *‘If government wants to address a health issue, something we agree to as an important matter to be given due and urgent attention, then it must utilize health related policy measures and not to use tax to dis-incentivise a particular lifestyle or to incentivise a particular health outcome, at the expense of existing economic benefits to the country*’ (Labour_2).

The general effectiveness of an SSB tax was debated by stakeholders. Opponents argued that SSBs weren’t a significant contributor to obesity, that physical activity was a better solution and that the tax wouldn’t work because consumers would substitute SSBs with other calorie-dense foods. Proponents argued that taxes would lead to reduction in consumption of SSBs with a significant impact on obesity. Others linked obesity with childhood undernutrition prevalent in Africa. *‘In many African countries, including South Africa, babies are given sugary drinks as a weaning food or even as a substitute for infant formula, which increases under-nutrition and stunting. Stunted infants have a much greater risk of becoming obese and diabetic’* (CSO_3).

##### Procedural concerns

Though not widely raised, procedural issues, about the law-making process, insufficiency of public engagement and others were used to challenge and delay the adoption of the tax. *‘Allow us to continue to contribute towards making a better South Africa by ensuring that the proposed tax on sugar sweetened beverages is not implemented. We do not believe it is economically and politically the right time to implement this tax’* (Industry_3).

Opponents of the tax argued that public engagement around the tax had been insufficient. Industry stakeholders argued that NEDLAC, a forum consisting exclusively of industry, labour and government, should have a larger role in the decision to adopt a tax and run its own participation process.

Many opponents of the tax also argued that a more comprehensive socio-economic impact assessment study, referred to as a SEIAS, was needed, even after government conducted a further SEIAS to address these concerns: *‘[a] SEIA[s] that does not meet necessary prescripts was provided to industry and Labour’* (Industry_3).

These arguments were often coupled with an express call to delay the implementation of the tax. *‘Why rushing to implement tax? Other measures are in place but are taking time to implement [association name redacted] not given adequate time to consider proposal. It is suggested by one sector that time should be allowed for reformulation’* (Industry Association_7).

Some proponents opposed delaying the implementation of the tax, arguing that inaction would have its own costs. *‘The implementation of the levy should not be delayed as 320 people are being diagnosed with diabetes on a daily basis and 10,000 cases of diabetes are being recorded every month in the country. South Africa is in a crisis and April 2018 might be too late. The opportunity to protect communities should not be missed’* (CSO_3).

##### Structure of the tax

There were specific proposals about the taxation structure, including the rate and scope of the tax from stakeholders. SSB and sugar Industry stakeholders generally did not comment on the tax rate, opposing the tax wholesale. However, fruit juice and concentrate industry stakeholders argued for lower tax rates, exemptions for their products or for only added sugars to be taxed. Some of proponents argued that fruit juices should not be exempt, as free sugar is also harmful. *‘Fruit juices and dairy -based drinks with naturally-derived caloric sweetener … may contain equal or higher levels of sugar than carbonated soft drinks’* (Health Associations_3).

Most proponents of the tax did not support the proposed rate, arguing for a higher taxation rate to achieve better health outcomes. Several proponents of the tax challenged the exemption for the first 4 grams per 100 grams of sugar, arguing that it reduced the effective rate of the tax and was not evidence-based. *‘There is no evidence that the benefits of this step in incentivising manufacturers to reduce the sugar content of some drinks, exceeds the disadvantage of leaving “less sugary” drinks untaxed. Indeed, allowing this exemption will threaten health promotion efforts favouring consumption of zero-sugar beverages (best of which is water)’* (Health Associations_3).

Varied arguments on how revenue from the tax should be used were raised. Some stakeholder questioned government’s motives for introducing the tax and whether health outcomes were the primary objective. *‘Global trend towards consumption and “stealth” taxes seems more to be a political choice for governments to obscure per person tax collections, as direct taxes are more visible and have become more unpopular and difficult to impose in a tax competitive global economy’* (Industry Association_1).

Proponents of the tax, specifically CSOs and academia, argued for the resulting revenue to be used for health-promoting activities. *‘Earmarking [of the revenue] is likely to increase the acceptability of the tax by politicians and the general public by increasing transparency over the taxation process and the use of revenues’* (CSO_2).

Labour stakeholders also supported the ring-fencing of the tax but argued the proceeds should be used to assist the sugar industry. *‘If the SSB tax were to come into force and its objective is to promote a healthier nation, then its revenue should be ring fenced initially to assist the sugar sector to transition to biofuel or other agricultural production with the condition that no worker will be retrenched’* (Labour_2).

### Influence of arguments on tax revisions

The two Treasury response documents were used to assess how policymakers responded to the stakeholder arguments and how these influenced the decision-making.

Over the duration of the policymaking process the SSB tax changed in four respects. First, the effective rate was reduced from 20% to 11%. Second, the exemption of the first 4 g of sugar/100 ml from the tax further reduced the effective rate. Third was reduction in the taxation of concentrates. Fourth, a higher tax rate was introduced for drinks which did not include sugar content on their packaging through a deemed sugar content of 20 g/100 ml. For the sake of clarity, it ought to be noted that the most significant of the changes – the effective rate and the introduction of the threshold – occurred before the parliamentary process and in response to the initial commenting process managed by Treasury.

Treasury grouped the comments and their responses into nine categories: scope of the proposed tax, proposed tax rate, proposed thresholds, tax revenue, administration, legislative mechanism, rebates and refunds, potential job losses, consultations process which traversed the six themes emanating from the submissions and meetings.

#### Economic considerations

Treasury was particularly responsive to arguments related to the economic impact of the tax, particularly job losses. *Treasury made several changes to the tax to reduce* its impact on the industry, including lowering the rate of the tax and introducing a threshold to exempt certain amounts of sugar. ‘The revised rate, and the introduction of the threshold, is based on the comments from stakeholders and is considered to be a critical part of the amended design of the tax to mitigate job losses … . The introduction of a threshold is part of the amended design to reduce the effective tax rate and mitigate job losses’ (Treasury Response_2). Treasury also used local evidence that an SSB tax was a cost-effective, population-level intervention to prevent NCDs to support its decision to adopt a tax.

Beyond this, Treasury also provided evidence to dispute claims about job losses and the negative economic impact of a tax, and explicitly stating that their own analysis showed less of an adverse economic impact than industry studies. *‘National Treasury modelled the potential impacts of the proposed levy … The net negative economic impact is significantly lower compared to studies commissioned by the beverage industry’* (Treasury Response_2). They also emphasised that the economic effects of the tax could be ameliorated through reformulation.

#### Impact on the vulnerable

There were no comments or responses from Treasury identified for this argument.

#### The responsiveness of an SSB tax to the problem of obesity

Although a number of submissions disputed the evidence in support of the effectiveness of an SSB tax for NCD prevention, Treasury anchored its decision in both the National Department of Health’s NCD Strategy and evidence from the WHO to support their view that an SSB tax would prevent obesity.

Treasury also confirmed SSB taxation would be part of a comprehensive package of interventions and was not a ‘silver bullet’ to address NCDs. *‘The implementation of the tax on sugary beverages is part of a comprehensive package of measures outlined in the Strategy and has not been put forward as the single policy response that will achieve the desired health outcomes*’ (Treasury Response_2).

#### The appropriateness of an SSB tax in South Africa

Treasury asserted that the implementation and enforcement of the SSB tax were feasible and were not administratively burdensome. On this basis, Treasury rejected a number of suggestions, including limiting the tax to added sugar and changing the design of the tax. ‘*A simplified tax regime (i.e. a single rate) is the most appropriate, in our view, and has administrative advantage compared to a multi bands and rates regime. The use of multiple tax bands adds to the administrative cost and enforcement burden of the tax’* (Treasury Response_2).

Treasury justified the focus on SSBs as the primary target of the tax due to their particularly harmful effects on health and evidence that consumption of SSBs was increasing. *‘Volumes of sugary beverages consumed are high and on the rise, and do not provide the same feeling of fullness that solid food provides. There is extensive scientific evidence supporting the contribution of sugary beverages to obesity, NCDs and oral health’* (Treasury Response_2).

#### Procedural concerns

In addressing procedural challenges, Treasury stated that the tax was ‘comparable to an excise duty’ and, consequently, did not require more public engagement. While they acknowledged the need for a participatory, Treasury emphasised that this should not delay the implementation of the tax.

Further Treasury stated that consensus could not be the threshold for a sufficiently participatory process. Treasury also cautioned that there should not be an over-reliance on the NEDLAC process as it would exclude ‘key players’ from the process. *‘Given that any tax proposal is subject to extreme lobbying, especially by those directly affected, it is almost impossible to reach agreement … . Such processes are also subject to undue delays, as affected stakeholders will benefit from any delay. It should also be borne in mind that in the NEDLAC process, key players may be excluded (such as trade unions like FAWU that represent workers in the beverage industry, as well as health experts and academics.’* (Treasury Response_2)

#### Structure of the tax

The structure of the tax was the chief concern of Treasury’s response documents and, as outlined above, there were a few changes to the tax in response to the comments. Responding to arguments for a higher tax rate, Treasury argued that a lower tax rate could still have a positive health impact. ‘*The studies do not show that the impact will only be realised with a tax rate above 20%. … However, there will be less of an impact than if the effective tax rate was set at 20%’* (Treasury Response_2).

Treasury justified the inclusion of the threshold for exempt sugar and the exemption of fruit juices on the basis that intrinsic sugar was not as harmful as added sugar, and that 100% fruit juices did contain some nutrients and thus required a different approach to other SSBs. In addition, Treasury outlined that these changes would simplify the implementation of the tax.

Treasury was particularly responsive to the issue of concentrates, which had been raised repeatedly by a producer, underscoring the need for the tax to apply equally to all categories of beverages within its scope. However, Treasury also recognised that producers might utilise this change to reduce the amount of tax payable on their products.

Finally, regarding the use of revenue, Treasury indicated that it would not be earmarked or ring-fenced for any particular purpose but that there was a commitment to utilise funds for health promotion programmes. *‘The legislative earmarking of revenue is not supported as it will introduce rigidities in the budgeting process. SA government has committed to increasing investments in health promotion targeting NCDs’* (Treasury Response_2).

## Discussion

The process of passing the SSB tax in SA was politically complex, with diverging views and contentions by stakeholders. Industry actors and associations, labour, academia and civil society were all key participants in the process. The types of positions adopted by stakeholders aligned with what has been observed in other jurisdictions such as Mexico, the United Kingdom and Philadelphia which adopted an SSB tax, namely that stakeholder positions were often reflective of their vested interests; and the links between the problem to be solved (obesity and NCDs) and the policy solution (SSB taxation) being a particular point of contention [6,19,25,26].

Often, health associations, academia and civil society supported the tax proposal, underscoring the health benefits of adopting a tax, while advocating for stronger and more comprehensive measures. This reflected the position in the United Kingdom where public health advocates were prominent voices in advocating for an SSB tax which culminated in the adoption of a tiered tax targeting sugar content in beverages [25].

Industry actors and labour were largely opposed to the adoption of the tax, arguing for the use of voluntary actions in lieu of a tax, disputing evidence in support of a tax, and overstating the economic harms caused by a tax. This combination of arguing for delaying implementation, attempting to dilute the policy adopted and delegitimising the evidence-based supporting the intervention reflects the industry ‘playbook’ used in other NCD prevention policies [25,27–29]. The strong emphasis on the economic impact of the tax, and resulting job losses, reflected similar rhetoric in other LMIC countries such as Mexico, Colombia and Brazil, where it was argued that a tax would cause significant job losses or other economic harms to the industry [6,20,26]. It appears that to a point, LMIC governments are particularly sensitive to arguments related to job losses. These arguments were partially successful and initially resulted in a lower effective tax rate. However, after an initial reduction in the rate, South African decision-makers remained firm in their intention to introduce a tax and would not dilute the rates further.

We also saw that industry actors attempted to move decision-making to forums where they would have exclusive access to policymakers, such as NEDLAC which permit only industry, labour and government to participate. Supporters of the tax, such as health organisations and civil society, are excluded from participation in these fora. This reflects another common industry tactic when it comes to interfering with NCD-related policies [30,31]. Though the tax was slightly diluted and delayed by industry actors, these arguments ultimately did not persuade decision-makers to stop implementation of the tax. Other studies have described the tactics adopted by industry actors during the adoption of the HPL, which align with our findings [10,31,32].

However, analysing decision-maker responses provided a means to understand which strategies were effective and how they may have influenced policy. Decision-makers were responsive, to a point, to industry claims regarding economic hardship. The response illustrated clearly how the tax was diluted through the lowering of the rate and the introduction of a threshold, to address industry concerns. This underscores the particular context of sub-Saharan Africa where economic development is often a priority that is in conflict with NCD prevention [7]. However, decision-makers were able to stave off further dilution of the rate by conducting their own analysis of the economic impact and may be a useful approach for other governments. Support from civil society and health organisations did not appear to receive much consideration from the policymaker, possibly because this was considered when initially developing the policy.

Although there was extensive debate about the evidence base supporting an SSB tax and the causes of NCDs in SA, Treasury deferred the Ministry of Health’s determinations on health impact. This underscores the importance of coordination between different ministries when passing cross-sectoral intervention measures, such as an SSB tax [33].

How revenue should be used was contentious both in the policymaking process and in other studies [16,17]. Proponents of the tax wanted revue to be directed towards health promotion activities, while opponents argued for the revenue to be used to offset the economic harms suffered by industry. The focus on government motives may be a result of the increased prevalence of corruption in government at the that time the policy was considered [34]. Research on the SSB tax adopted in Philadelphia indicates that, while outlining the health benefits of a tax was important, transparency in revenue use was a critical issue in developing support [35]. A further, context-specific concern that emerged from the policymaker’s perspective was the feasibility of implementing a tax, which had a substantial impact on its design and structure. This is relevant for other SSA countries considering adopting an SSB tax.

Arguments contending that measures like SSB taxes impinge on individual autonomy and promote a nanny state often emerge in high-income countries like the United States and the United Kingdom [19,36]. However, these types of arguments were not prominent themes in the submissions. There was consensus from all stakeholders, even those opposed to the tax, that action was needed on NCDs, and all actors supported government taking action on NCDs.

### Strengths and limitations

This was a desk-based review which relied on publicly available information; there are inherent limitations and strengths with this approach. The review enabled us to capture arguments as framed by the stakeholders themselves and was not dependent on their willingness and availability to participate, or on recollection of events. The utilisation of committee meeting recordings and policymaker responses enabled us to present a holistic picture of the formal policymaking process from the perspectives of both the public and the decision-making bodies. However, this approach enabled us to consider only a limited aspect of the policymaking process for the SSB tax, namely the formal and recorded parliamentary process. Informal, extra-parliamentary activities that influenced the process were not captured. Future research may consider examining the informal policy process, as done in other countries, through other social science methodologies and from data sources such as media analysis and political economy analyses.

## Conclusion

Arguments by both the proponents and opponents of the HPL influenced the policy that was adopted. The decision-maker sought to ameliorate adverse economic consequences of the tax by lowering the tax rate and revising the structure, but these arguments were critically considered and successful only to a point. Pro-tax arguments, specifically on the health impact of the tax, buttressed the intention to adopt a tax. The arguments used to advocate for or against an SSB tax and NCD prevention policies in SA were broadly consistent with those used in other countries, although there are some context-specific nuances that should be taken into account by those seeking to implement an SSB tax.

## Paper context

Taxation of sugary beverages is often controversial. The adoption and form of taxes may be influenced by a variety of stakeholders. This paper analyses the participation of different stakeholders in the process of adopting such a tax in South Africa to understand their influence on the final policy. We found that the policy was influenced positively and negatively by stakeholders with the financial and health impact of the tax being the most influential arguments advanced.
